# Simulating Dilute-Solution Properties and Behavior of Flexible Macromolecules: A Review of Brownian Dynamics, Monte Carlo Methods, and Computational Tools (SIMUFLEX and MONTEHYDRO) with Applications to Biomacromolecules and Selected Synthetic Polymers

**DOI:** 10.3390/ijms27177791

**Published:** 2026-08-31

**Authors:** José García de la Torre, José G. Hernández-Cifre

**Affiliations:** Departamento de Química Física, Facultad de Química, Universidad de Murcia, 30100 Murcia, Spain; jghc@um.es

**Keywords:** Monte Carlo simulation, brownian dynamics simulation, macromolecular modeling, solution properties, internal dynamics, single-molecule behavior, DNA, ultracentrifugation, dendrimers, drug delivery

## Abstract

Dilute-solution properties are important sources of information on the structure of macromolecules. Analyzing experimental data and extracting information on structural properties require theoretical and computational resources. The resources needed to study rigid particles are manageable; however, studying flexible particles is more challenging. This is because, unlike rigid bodies, and as a consequence of the conformational variability arising from flexibility, flexible particles do not have a definite size and shape. In addition to their overall translational and rotational Brownian motion, the dynamics of flexible particles in solution has an internal component: size/shape conformational fluctuations. In order to facilitate the study of flexible macromolecule hydrodynamics, we have implemented existing theories within several computer programs. MONTEHYDRO combines Monte Carlo simulations based on the importance-sampling algorithm to generate conformations from which, in addition to conformational quantities, the hydrodynamic properties of flexible particles can be obtained using rigid-body treatment. SIMUFLEX is a suite based on a Brownian dynamics simulation of macromolecules, comprising BROWFLEX, for the generation of trajectories, and ANAFLEX, for the calculation of static and time-dependent properties as well as the simulation of single-particle events. In this paper, we present some concepts which are fundamental to the methods implemented in those computational tools, as well as examples of their utilization in various biomacromolecule applications, with a particular emphasis on double-stranded DNA in various cases: coarse-grained double-helical models for moderately short DNA; the worm-like model treatment of DNA over an extremely wide range of sizes (from 8 to 200,000 base pairs); and the problem of the anomalous rotational-speed dependence of the sedimentation coefficient of very long DNA. SIMUFLEX has also been particularly useful for studying intrinsically partially disordered proteins, whose structure comprises both ordered, globular domains as well as flexible tail and linker chains. To illustrate applications in the field of synthetic polymers, we describe a study on dendrimers, with aspects related to drug delivery in targeted therapies.

## 1. Introduction

Since the seminal work of Einstein, the properties of molecules in solution have become essential sources of information on macromolecular structure [1,2]. Since the advent of the concept of macromolecules, many methods have been widely employed to determine their sizes and shapes or conformations, including dilute-solution properties; techniques like analytical ultracentrifugation, which have been applied to colloids and biomacromolecules [3,4,5]; and solution viscosities for studying polymers [6,7].

Over time, theories and methods have been developed to co-relate experimental data and structural aspects. For rigid macromolecules (particularly some biomolecules) and nanoparticles that have a specific, well-defined size and shape, the field is well established; however, the case of flexible particles is still subject to further developments, as we described in previous papers [8,9]. Flexible particles are able to adopt a variety of instantaneous shapes (conformations); this variability introduces notable difficulties in theory and methodology, such as introducing statistical and hydrodynamic aspects. Furthermore, dynamic descriptions must consider not only the dependence of global properties (hydrodynamic coefficients, scattering-related properties, etc.) on the size and shape of a rigid particle, but the interconversion between the various conformations of a flexible particle, i.e., its *internal dynamics*. Thus, the internal dynamics must be considered, not only because it affects the overall motion, but because it determines many functional aspects and is currently widely studied to describe single-molecule events.

Modern theories comprehensively describe the fundamental aspects that govern both the overall and the internal dynamics of flexible macromolecules. However, the abovementioned difficulties make direct calculations based on explicit theoretical expressions challenging or even unfeasible. The current approach comprises computer modeling based on first principles. Classical Monte Carlo (MC) simulation facilitates the complete generation of the possible conformations of a flexible particle and provides a rigorous method for calculating non-dynamic properties, such as those observed during static scattering of light or X-rays, as conformational averages. In terms of the so-called rigid-body approximation, the Monte Carlo rigid-body method (MC-RB) provides relatively accurate predictions for the hydrodynamic coefficients of diffusion, sedimentation, and viscosity.

Brownian dynamics (BD) simulation is a truly rigorous approach for all types of molecular dynamics of macromolecules in solution, and is valid for a wide range of time scales including overall motion and internal dynamics. Based on the fundamental principles of the Brownian motion of particles in a continuous, viscous solvent, it provides a correct description of dynamic scattering and predicts single-molecule behavior. MC and BD simulations can both be implemented with the same coarse-grained model, force field, and scheme for the hydrodynamic interaction (HI) effects employed in rigid-particle bead-modeling hydrodynamics [10,11,12].

Over the past five decades, we have developed theoretical formalisms and procedures for MC and BD simulations, and applied them to numerous macromolecular systems. To this end, we have devised computational methods, which have resulted in publicly available computer programs including the SIMUFLEX and MONTEHYDRO suites. These tools enable simple and user-friendly predictions of the equilibrium and hydrodynamic properties of macromolecules, as well as their behavior in solution at the molecular level.

In this article, we describe the implementation of BD and MC methodologies in our tools, which allow for the rigorous and effective prediction of the dilute-solution properties of flexible macromolecules for structural information extraction. We first present a brief introduction to the methodologies, emphasizing how both methods consider the effect of intramolecular hydrodynamic interactions, which is fundamental to explaining the properties of an individual molecule in solution. This effect is not considered in other approaches that are not specifically oriented towards dilute-solution properties. Rigid-body hydrodynamics is integrated into MONTEHYDRO. Procedures based on the Ermak–MacCammon BD algorithm, which ignores inertia but thoroughly describes hydrodynamic friction and diffusion, as described below, are at the core of BROWFLEX.

A notable feature of the two programs is that they both share the same mechanical bead-and-spring model, which is a natural extension of the bead models employed in rigid-body hydrodynamic calculation schemes used in our HYDRO suite. The fact that the same model can be used interchangeably for MC and BD is useful. Thus, one can, for example, choose MC when considering conformational properties, such as those related to scattering, and accept the MC-RB results for global hydrodynamic properties, or choose BD simulation to obtain rigorous results of dynamic properties, especially those related to the internal dynamics of flexible macromolecules. The mechanical model includes a variety of options to specify the force/potential field of the model, the most important of which are described below. Next, we describe some features of the software, which has been programmed to be extremely easy to use, as has been the case in other programs developed by our group.

Our article concludes with sections that present a series of applications that we have selected for their relevance in the fields of biomacromolecules and synthetic polymers. Regarding the former, we focus primarily on applications to the most important macromolecule in the biosciences: DNA. We also include the case of a class of biomolecules of great current interest: intrinsically disordered proteins. Finally, to present a case from the field of polymers, we consider dendrimers, regular hyperbranched polymers with an extremely peculiar topology, which makes them useful in many applications. In the field of biosciences, these applications include the encapsulation of drugs for drug delivery, which is essentially determined by the internal Brownian dynamics of the dendrimer molecule.

## 2. Simulation Methods

### 2.1. Brownian Dynamics (BD) Simulation

The motion of a rigid particle immersed in a viscous fluid can be described in terms of two aspects. If a constant force F is acting on the particle, one of the aspects is the deterministic displacement related to the fluid of viscosity $\eta_{0}$. For the particular case of a spherical particle, under this force the velocity of the particle (the time-derivative or its position vector **r**) will be v≡dr/dt=(1/f)F, where $f=6\pi \eta_{0}a$ is the Stokes law frictional coefficient of a sphere of radius *a*, related to the diffusion coefficient by the Einstein relationship $D=k_{B}T/f$ [2], where $k_{B}$ is the Boltzmann constant and *T* is the Kelvin temperature. In a time interval Δt, the displacement will be v·Δt. The other aspect arises from Brownian motion; according to the Einstein theory [1], the displacement of the position vector r during Δt is a random vector R(Δt) whose 3 components, (x,y,z), are random numbers with a Gaussian distribution with zero mean and covariance 2DΔt. Then, after the time step Δt, the final position r will be

(1) $$r=r^{0}+\frac{D\Delta t}{k_{B}T}F^{0}+R\left(\Delta t\right)$$

where the ^0^ superscript denotes the value at the initial position. The algorithm expressed by Equation (1) is the basis for simulating the Brownian trajectory of a single spherical particle.

For a system of *N* spherical particles, the situation is far more complex, mainly due to the so-called hydrodynamic interaction (HI) effect. The motion of each sphere is related to the positions and velocities of all the other spheres. The time evolution of the system is determined by a matrix, the diffusion tensor, D, of dimension 3N×3N. In a seminal paper, Ermak and McCammon [13] presented a generalization of Equation (1) for a multi-particle system. The Ermak–McCammon algorithm is expressed as follows:

(2) $$r_{i}=r_{i}^{0}+\frac{\Delta t}{k_{B}T}\sum_{j=1}^{N}D_{ij}^{0}\cdot F_{j}^{0}+\Delta t\sum_{j=1}^{N}{\left(\frac{\partial D_{ij}}{\partial r_{j}}\right)}^{0}+R_{i}^{0}\left(\Delta t\right)$$

The first term added to the initial position vector $r_{i}^{0}$ of the *i*-th sphere is the deterministic part of the displacement, which replaces the term $D\Delta t/(k_{B}T)$ in Equation (1). $D_{ij}$ is a 3×3 block of the matrix D. The next term arises from the HI effect, and disappears when the modified Oseen tensor [14,15,16] is used for the diffusion tensor. Finally, the Brownian displacements of the elements, $R_{i}\left(\Delta t\right)$, are such that their variance/covariance is given by

(3) $$\langle R_{i}\left(\Delta t\right)R_{j}\left(\Delta t\right)\rangle =2\Delta tD_{ij}^{0}$$

The above discrete, step-wise algorithm (Equation (2)) assumes that the forces $F_{j}^{0}$ and diffusion tensors $D_{ij}^{0}$ remain nearly unchanged during the time step Δt, which therefore, must be sufficiently small. In order to produce a sufficiently long trajectory, the number of steps will be relatively large. Furthermore, the computer calculations require a CPU time that scales as $N^{3}$. Several strategies have been proposed to overcome these difficulties. Our group [17] proposed a modification to the Ermak–MacCammon algorithm which implements procedures allowing for longer Δt [18]. Also, alternative ways to handle the diffusion tensors have been presented [19,20,21,22,23].

### 2.2. Monte Carlo (MC) Simulation

The generically named Monte Carlo simulation methods are formulated to generate a large sample of possible conformations of a multi-particle system. The observable properties are calculated as averages over the individual values for the conformation. The statistical weight for each conformation in the sample is proportional to the Boltzmann exponential of the potential energy, $V_{tot}\left(q\right)$, which is evaluated from a suitable set of coordinates, q, of every particle as follows:

(4) $$w\propto \exp\left(-\frac{V_{tot}\left(q\right)}{k_{B}T}\right)$$

If the particles are spherical, or assumed to be point-like, q will be just a 3N vector containing the position vectors $r_{i}$ of the *N* particles, and $V_{tot}=\sum_{i}V_{i}$. The generation of possible conformations of the system can be completed using any conveniently devised, random sampling method.

In the original Monte Carlo method (developed during World War II through the Manhattan Project) the so-called “importance-sampling” procedure was a step-wise algorithm in which another conformation of the system, with coordinates q, is obtained from a previous one, which had coordinates $q^{0}$, adding a randomly generated displacement, Δq, to obtain $q^{\prime }=q^{0}+\Delta q$. If the potential energy is decreased, i.e., $\Delta V=V_{tot}^{\prime }\left(q^{\prime }\right)-V_{tot}^{0}\left(q^{0}\right)<0$, the new conformation is accepted. Otherwise, an acceptance decision is taken: a uniformly distributed random number, *u*, is generated and the conformation is still accepted, with $q\;=q^{\prime }$, if $u<\exp[-\Delta V/\left(k_{B}T\right)]$; otherwise, it is rejected, with the new conformation taken as a copy of the previous one so that $q=q^{0}$.

Like the choice of time step, Δt, in Brownian dynamics, the choice of the range of random displacement, Δq, in Monte Carlo simulation is somewhat critical, as it represents a compromise between two extreme situations. If Δq is too large, the probability of the new conformation being rejected is very high. Conversely, if Δq is too small, the new conformation will almost certainly be very similar to the previous one. In both extreme cases, a very large number of steps is required to sufficiently scan the model’s conformational space, resulting in a high CPU computation time, which is proportional to the number of steps. Thus, in both MC and BD simulations, preliminary trials are needed to find the optimum step size.

In this procedure, Boltzmann statistics are taken into account *a priori* when generating conformations, and the observable properties are simple, non-weighted averages. It has been demonstrated that the importance-sampling procedure is far more efficient than simple procedures. The paper from Metropolis et al. [24] was the first to publish this method.

The instantaneous conformations in the trajectories generated using Brownian dynamics constitute a correctly weighted sample, like in the Metropolis Monte Carlo method. This is true even if the computationally expensive hydrodynamic interaction effect is neglected (non-HI BD) [25] and may be an alternative to and, in many cases, more efficient than MC simulations.

## 3. Mechanical Model and Force/Potential Fields

### 3.1. Beads and Springs

Both Monte Carlo and Brownian dynamics simulations can be applied to any multi-particle system. Our methods target the properties and dynamics of a single molecule, as they apply to macromolecules in a dilute solution. Firstly, the model is composed using *N* elements, which can represent whichever kind of molecular constitutive entities are considered in the model: atoms, residues, monomeric units, subunits, partial regions or subchains, etc. Then, the primary inter-particle interactions are connectors between the beads that describe the molecular topology and have features that somewhat represent the chemical structure. The elements in such an array of elements are assumed to be spherical (or even point-like), and, when representing dynamic aspects, they are immersed in a continuous viscous fluid, the solvent.

This view was the basis of the bead-and-spring model proposed in the pioneering theories for linear macromolecules in solution. The famous bead-and-spring model proposed by Rouse [26] and further developed by Zimm [27] comprises a chain of beads where the connectors are Hookean springs with a linear force and a quadratic potential, which as shown as follows:

(5) $$F=-Hlu_{l}\;V=\frac{1}{2}Hl^{2}$$

where *H* is the Hooke force constant, *ℓ* is the instantaneous length, and $u_{l}$ is the unitary vector along the spring direction. The quadratic potential energy provides Gaussian statistics for *ℓ* as it corresponds to a random-flight model for the end-to-end distance of the molecular subchains, with a probability function and a mean square value calculated as follows:

(6) $$p\left(l\right)\propto \exp\left[-\frac{V(l)}{k_{B}T}\right]\;\langle l^{2}\rangle =\frac{3k_{B}T}{H}$$

Elementary descriptions of the Rouse–Zimm model can be found in textbooks [28,29,30].

**Figure 1:**
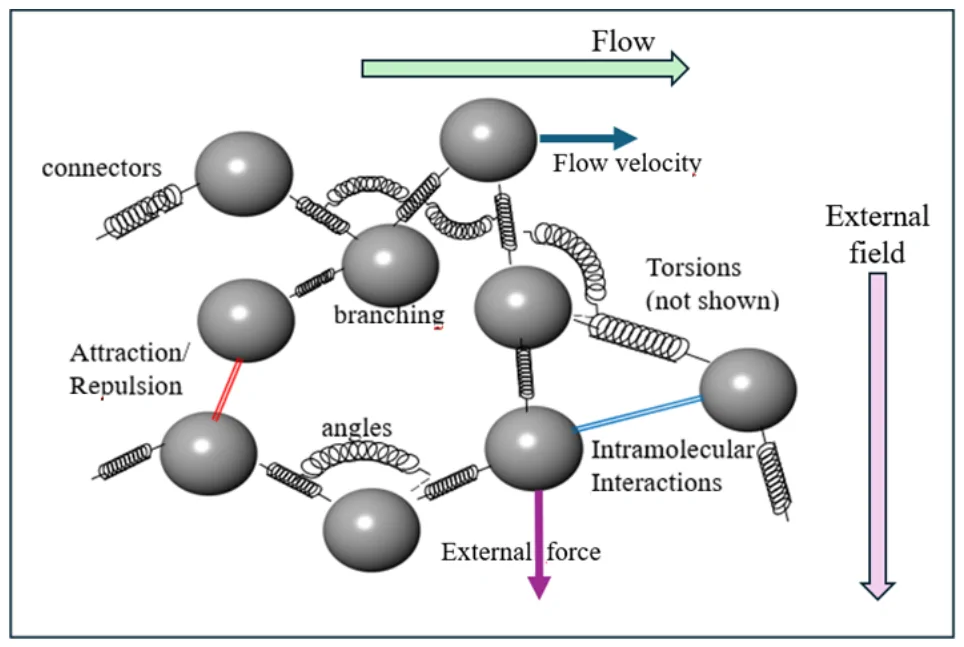
Illustration of the mechanical model, indicating some components that it can contain. The arrows indicate the possibility of applying external interactions (flows, force fields, and/or forces acting on specific elements of the model) to the bead-and-spring model.

Inspired by this classical model, our SIMUFLEX and MONTEHYDRO programs consider a general mechanical model, composed of *N* spherical beads joined by spring-like connectors, which experience frictional forces in a viscous solvent, intramolecular interactions, and, eventually, the action of external fields or flows. A schematic image is displayed in Figure 1.

Thus, the mechanical model is somewhat of an extension of the bead–model hydrodynamic representation of rigid particles, which are modeled as rigid arrays of beads, a model which has been successful in the representation of rigid biomacromolecules and nanoparticles [8,9,31]. Rigid bead models can be used even for atomic/residue-level calculations for rigid proteins and nucleic acids [12,32,33].

The bead modeling paradigm forms the basis of our mechanical model. In MC and BD simulations, beads represent individual atoms (eventually, they can represent extended/united atoms, including hydrogen) such as in conventional molecular dynamics simulations; some examples can be found in refs. [34,35,36]. However, commonly used procedures employ a coarse-grained model of a macromolecule as an array of connected beads, which corresponds to certain portions of the macromolecule, ranging in size from a few atoms—e.g., a monomer, residue, etc.—to a large portion, say a subunit, subchain, etc. In our MC and BD procedures, the topology of the connected array is arbitrary. In addition to the obvious case of linear chains, our programs can be applied to a variety of macromolecular architectures such as circular, ring-shaped DNA and synthetic polymers [37,38,39,40,41,42]; branched chains, [43,44] including star-like [45,46,47,48], comb-shaped [49], and hyperbranched polymers; and regularly branched [50,51,52] or randomly branched dendrimers [53,54]. These simulation methodologies may be applicable to polymers with even more complex topologies, like brushes in which dendritic branches are grafted onto a linear skeleton or a planar surface [55,56].

The force/potential functions of the spring-like connectors depend on the nature of the chemical entities that they represent. Our programs offer a number of possibilities that are adapted to the different scales that may be represented by the mechanical model. When one program is used to model very long and flexible, high-molecular-weight macromolecules, such as synthetic polymers and native DNAs, the elements will typically represent long flexible subchains that obey a Gaussian statistic for the components of an end-to-end vector, and the springs could be described, like in the Rouse–Zimm model, by the Hookean force and Gaussian potential (Equation (5)). The value of the Hookean constant is taken such that mean square elongation corresponds to the mean square end-to-end distance of the subchain, i.e., $H=3k_{B}T/\langle l^{2}\rangle$ according to Equation (6).

A chain of Gaussian, Rouse–Zimm springs has a non-physical feature, the zero equilibrium length, with V=0 for l=0. A convenient modification is the so-called Fraenkel spring [57], for which the potential is still Gaussian, with a non-zero equilibrium length, $l_{e}$, with $V(l_{e})=0$, and the force is still Hookean, shown as follows:

(7) $$F=-H\left(l-l_{e}\right)u_{l}\;,\;V=\frac{1}{2}H{\left(l-l_{e}\right)}^{2}\;,\;p\left(l\right)\propto \exp\left[-\frac{1}{2}\frac{H}{k_{B}T}\delta_{l}^{2}\right]\;,\;\delta_{l}=l-l_{e}$$

The force constant determines the fluctuation in the spring length; from the Boltzmann probability function in Equation (7), the relative root mean square (rms) deviation from the equilibrium length is

(8) $$\delta_{l}^{*}\equiv \frac{<\delta_{l}^{2}{>}^{1/2}}{l_{e}}={\left(\frac{3k_{B}T}{l_{e}^{2}}\right)}^{1/2}$$

In an atomic-scale dynamic simulation, a Fraenkel spring with a sufficiently large value of *H*, and therefore, a relatively small length fluctuation, is a more convenient representation of a chemical bond than a fixed bond (a rod in the model) because it avoids the mathematical difficulties caused by the constraint. It is particularly useful for residue-level coarse-grained simulations with SIMUFLEX and MONTEHYDRO that represent virtual bonds in models where the elements correspond to small portions of the molecule; for instance, repeating units in polymers or amino acid or nucleotide residues in residue-level simulations of biomacromolecules, as described below for DNA [58] and proteins [8,59].

An important extension of these simple quadratic potentials is considering a maximum value for the spring length, $l_{max}$. This can be applied, with the same formalism, to two different situations. One scenario is describing the conformation and dynamics of very flexible random-coil chains in strong shear or similar flows or under external forces; the flexible subchain represented by a spring may approach a fully extended length conformation $l_{max}$. Another scenario is that of small molecular entities with some degree of flexibility, whose fully extended length is not greater than the typical mean values.

These cases are well described by the finitely extensible nonlinear elastic (FENE) spring whose original version, usually attributed to Warner [60], was mainly intended for the first of the two mentioned situations. The FENE force and potential are given by

(9) $$F=-\frac{Hl}{1-{\left(l/l_{max}\right)}^{2}}\;V=-\frac{1}{2}Hl_{max}^{2}ln\left[1-{\left(l/l_{max}\right)}^{2}\right]$$

It can be easily verified that when *ℓ* is much smaller than $l_{max}$, with $l/l_{max}<<1$, the expressions in Equation (9) tend to those for the Hookean, Rouse–Zimm spring in Equation (5). On the other hand, when *ℓ* approaches $l_{max}$, both *V* and −F take very large values. Thus, the spring length is limited to the range $0\leq l\leq l_{max}$. Pamies et al. [61] proposed a procedure for parameterizing FENE models of long, flexible chains with data for the values of the maximum elongation and the radius of gyration of the chain in the absence of external agents.

**Figure 2:**
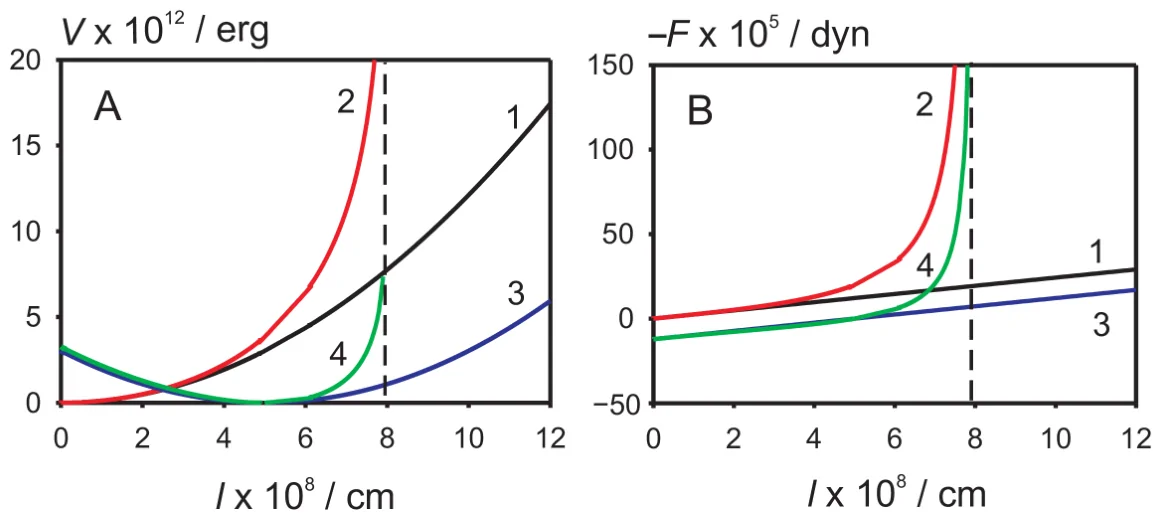
(**A**) Length dependence of the potential energy of a spring. (**B**) Length dependence of the force of a spring. The types of springs and spring parameters used to build the plots in both (**A,B**) are: (1) Gaussian spring with H=2424 erg/${\mathrm{cm}}^{2}$. (2) FENE (Warner) spring with H=2424 erg/${\mathrm{cm}}^{2}$ and $l_{max}=8\times 10^{-8}$ cm. (3) Hard Hookean (Fraenkel) spring with H=2424 erg/${\mathrm{cm}}^{2}$ and $l_{e}=5\times 10^{-8}$ cm. (4) Hard-FENE spring with the same three values of the parameters *H*, $l_{max}$ and $l_{e}$. These are typical values for the repeating units, i.e., the branches of dendrimer molecules [52]. The dotted line indicates the maximum length of the FENE-type springs.

The concept of finite extensibility is, of course, applicable to chains that have a non-zero equilibrium length $l_{e}$, with $V(l_{e})=0$. The so-called FENE-Fraenkel spring [62,63] includes an equilibrium length, as the Fraenkel spring, and a maximum length, $l_{max}$, with $V(l\to l_{max})\to \infty$, as the FENE spring. The FENE-Fraenkel spring is thus defined by three parameters, *H*, $l_{e}$, and $l_{max}$. Other three-parameter potentials have been proposed in the literature: the Morse potential, [64,65], which is well known for describing chemical bond dissociation in diatomic molecules [66]; and the hard-FENE potential [52,67], which was devised for cases when $l_{max}$ is not much larger than the equilibrium length. Details can be found in the cited literature. Figure 2 displays the dependence of the force and potential for four kinds of springs with and without an equilibrium length and with and without a maximum length.

Let us now consider the number, *N*, and size of the model elements. The number of them should be moderate (in some cases, up to 100), since a full treatment of the hydrodynamic interaction (HI) requires calculations with a CPU time proportional to $N^{3}$. Thus, the mechanical model for large macromolecules should be more or less coarse-grained.

Regarding the size of the spherical beads, which are needed to calculate hydrodynamic properties, their radius, ζ, should be taken as the hydrodynamic radius [8,9], $\zeta \equiv R_{H}$, of the entities that they represent. Recall that $R_{H}$ is obtained from the friction or diffusion coefficients, *f* and *D*, respectively, as

(10) $$R_{H}=\frac{f}{6\pi \eta_{0}}\;\mathrm{or}\;R_{H}=\frac{k_{B}T}{6\pi \eta_{0}D}$$

When simulating very long flexible macromolecules, the elements represent long flexible subchains whose hydrodynamic radius is related to conformational properties, like the mean square end-to-end distance $\langle r^{2}\rangle$. The radius of gyration is given by $R_{g}^{2}=\langle s^{2}\rangle /6$. In the mechanical model, the springs should be parameterized so that the elongation $\langle l^{2}\rangle \equiv \langle r^{2}\rangle =6R_{g}^{2}$. For flexible, random-coil chains, the radius of gyration and the equivalent radii are related as $R_{H}=R_{g}/GT$, where GT is an universal constant [8,9,68] whose value is 0.78 in ideal conditions (“theta” solvent) or, what is more usual, 0.69 in good solvent conditions, as in the case of biomacromolecules in aqueous media. Thus, in these cases, some previous knowledge or separate calculations of conformational properties would be employed to parameterize the bead-and-spring models.

In models where elements represent small chemical entities (residues, monomeric units, etc.), structural analysis and rigid-body hydrodynamics at atomic resolution [12,69] might be useful to estimate their hydrodynamic radius.

### 3.2. Other Intramolecular Interactions

In many cases, the internal structure and dynamics require the introduction of forces (potentials) associated with the angle, α, subtended by two contiguous springs (like bond angles in conventional molecules). For many cases, a quadratic bending potential with an equilibrium value $\alpha_{e}$ is adequate:

(11) $$V=\frac{1}{2}K_{\alpha }{\left(\alpha -\alpha_{e}\right)}^{2}$$

The forces acting on the three beads—with the expression omitted here—are properly evaluated from the gradient of potential. Since the angle potential involves two consecutive connectors, joining three connected beads, another torsional potential, corresponding to three connectors and four beads, may be required in other cases.

In addition to these short-range intramolecular interactions, there may be others corresponding to elements that, although distant along the connectivity, can be instantaneously close in space. These kinds of interactions represent the excluded volume (EV) effect. The potential associated with a pair of elements i,j will depend on the distance, $r_{ij}$, between them. There is a great diversity of expressions for the functional form of the dependence. One of them is the well known Lennard–Jones potential, which includes an attractive term and a repulsive one [66]:

(12) $$V\left(r_{ij}\right)=4\epsilon_{LJ}\left[{\left(\frac{\sigma_{LJ}}{r_{ij}}\right)}^{12}-{\left(\frac{\sigma_{LJ}}{r_{ij}}\right)}^{6}\right],$$

where $\epsilon_{LJ}$ is the maximum attractive energy and $\sigma_{LJ}$ is the distance at which the attractive and repulsive term cancel to each other and when V=0. Another very simple and yet useful pairwise potential is the hard-spheres potential, given by $V(r_{ij})=0$ (no interaction) if $r_{ij}\geq \sigma_{HS}$ and $V(r_{ij})=\infty$ if $r_{ij}<\sigma_{HS}$, where $\sigma_{HS}$ is taken as the sum of the two geometric radii of the spherical beads.

(13) $$V\left(r_{ij}\right)=\left\{\begin{array}{cc} 0 & r_{ij}\geq \sigma_{HS}\\ \infty & r_{ij}<\sigma_{HS} \end{array}\right.$$

The mechanical model can include other types of intramolecular interactions and external agents (confinements, electric and flow fields, etc.), which are detailed in the user guides of SIMUFLEX and MONTEHYDRO.

## 4. Computer Programs

Both MONTEHYDRO and SIMUFLEX are programs that interact with the user simply by means of computer input and output text (*.txt) files. The input files of both programs first contain some basic information about the mechanical model as follows:

- The initial conformation from which the simulation will be started, specified by the number of beads and a list with one line for each bead. Each line contains the values of the Cartesian coordinates, $(x_{i},y_{i},z_{i}),$ and the hydrodynamic radius, $\sigma_{i}$, of the bead *i*.
- The number of bonds between beads, which is represented in the mechanical model by springs, and a list with one line per bond specifying the indices of the pair of connected beads, (i,j), the kind of spring (Hookean, Fraenkel, FENE, etc.) and the spring parameters.
- The number of angular, bending interactions involving three beads, (i,j,k), such that the pairs (i,j) and (j,k) are bonded, with *k* being the central bead. A list is provided, with one line for each angle, giving the triad (i,j,k), along with the type of force/potential and its corresponding parameters.
- The number of torsional interactions (measured by dihedral angles) involving four beads, (i,j,k,l), such that the pairs (i,j), (j,k), and (k,l) are bonded, with (j,k) being the central, rotatable bond. A list is provided, with one line for each angle, giving the tetrad (i,j,k), along with the type of torsional force/potential, and its corresponding parameters.
- Non-bonded intramolecular pair interactions, involving pairs of non-bonded beads. A very frequent case is that of excluded-volume, steric effects, simply represented by a limit in the inter-bead distances or, more generally, attractive–repulsive van der Waals-type interactions and similar; our force field includes a variety of types.
- For both programs, the user specifies a simulation step size, which is the time interval between two successive conformations, based on Brownian dynamics, or the range of the displacements between conformations during importance-sampling Monte Carlo simulation. The physical simulation time is determined by the number of time steps of that size (and the CPU computing, as well). A key requirement is that the time step is as small as possible, which means that the number of steps is quite large. Only a fraction of the simulated conformations can be registered in the trajectory; the number of them is specified in the input file.
- The temperature should also be given for both programs.

The core of the output generated by both programs is a series of successively recorded conformations. In Brownian dynamics that consider hydrodynamic interaction effects (HI), a true trajectory in real physical time will be produced, i.e., a succession of records with particle coordinates and other information at successive instants during Brownian motion. In Monte Carlo simulation, a collection of conformations simply serves as the sample for statistical analysis and should not be mistaken for a physical time trajectory. The successively recorded conformations are required for any further property computation or analysis.

### 4.1. The MONTEHYDRO Package (For MC-RB)

The MONTEHYDRO input file contains data corresponding to other features. The model may contain beads that do not move in the simulation, which may be useful for simulating systems with attachments, obstacles, cavities, etc. The input file refers to a separate file, where the position and sizes of the still, stationary beads are given.

The main output file contains a sample of instantaneous conformations generated using simulations. As described in the user guide, this file contains a series of blocks, with each one corresponding to a conformation and containing the coordinates of the beads. Users can readily access this file for their own processing; the usual procedure is to read these blocks sequentially, evaluating the properties under study, to finally produce simple averages over the conformations. We recall that such averages will be unweighted, since the importance-sampling MC algorithm was employed in the simulation.

Nonetheless, the execution of MONTEHYDRO itself includes the evaluation of significant, average properties, which may be useful to confirm the correctness of the simulation. In the default case, these will be the mean square end-to-end (from bead 1 to bead *N*) distance, $\langle r^{2}\rangle$, which is a relevant property for models with a linear topology, and the radius of gyration, which is applicable to arbitrary topologies. Obtaining these quantities directly from the simulation may be useful for testing. Another option is that the execution will yield two additional basic hydrodynamic properties: the diffusion coefficient and the intrinsic viscosity (having previously provided data for the solvent viscosity, $\eta_{0}$, and the molecular weight, *M*). The program adopts the rigid-body Monte Carlo (RB-MC) scheme [70,71,72], producing a rigid-body calculation of these properties for each conformation—the program’s core includes the code of our HYDRO++ program [73], which is the most general and advanced procedure for rigid-body bead models.

### 4.2. The SIMUFLEX Package (For BD): BROWFLEX and ANAFLEX

The first program in this package, BROWFLEX, considers Brownian dynamics simulations. Unlike MONTEHYDRO, its almost-exclusive function is trajectory generation. The various and numerous modes for trajectory analysis and property calculation are grouped within ANAFLEX.

BROWFLEX shares many of the abovementioned aspects of the force field and common data with MONTEHYDRO. The volume of data is larger and is organized into a few data files with specific content: content related to the molecular model; the simulation parameters, options, and conditions; content that might correspond to flows, electric fields, and other external agents in a separate file, etc. The primary result is a simulated trajectory, that is, a succession of conformations that the system adopts throughout the simulation, as in MONTEHYDRO, which is then presented as a true real-time trajectory.

ANAFLEX reads the generated trajectory and performs various types of calculations and analyses. One very simple step is calculating conformational averages, of the steady-state type, of properties in a manner analogous to MONTEHYDRO, i.e., averaging properties evaluated for successive conformations and applying the rigid-body treatment. The conformational sampling performed using BD, like that of MC, implicitly involves importance-sampling, and the averages will be the arithmetic mean. To perform real-time simulations, such as those indicated below, it is essential to activate the hydrodynamic interaction (HI) effects, but it is interesting to note that simulations that do not include them correctly sample the conformational space, so BD-noHI can be an efficient alternative to MC.

Another relevant application of BD involves properties closely related to Brownian motion, which are calculated using first principles. This includes the complete simulation of diffusion, reorientation dynamics, and internal dynamics phenomena, which are particularly important for flexible particles. These phenomena are expressed as diffusion tensors, molecular relaxation times, and nuclear magnetic resonance, and are derived from time correlation functions. ANAFLEX incorporates a significant number of calculations of these types of quantities.

However, a valuable application of BD simulations is the prediction (simulation, visualization, and analysis) of single-molecule events. Detailed knowledge of how an individual molecule behaves can be achieved in quiescent solution, but also under the action of forces, fields, flows, etc. A paradigmatic example is the behavior of dsDNA under the action of intense shear or elongational flow, under which it undergoes stretching that causes it to adopt very peculiar stretched conformations, as observed experimentally by Chu and colleagues [74,75,76]. BD simulation reproduces every detail of the observations very well [67]. A related case is that of the anomalous sedimentation of very high-molecular-weight dsDNA, which undergoes stretching in a centrifugal field that influences the measured value for the sedimentation coefficient. Furthermore, ANAFLEX contains an interface with other programs of ours: VisualBeads for producing snapshots and MovieBeads for producing a video that explicitly shows single-molecule behavior. The movies included in the Supplementary Information were created using the latter.

## 5. Applications to DNA

After the historical Watson–Crick paper [77] that described the molecular structure of DNA based on their analysis of the X-ray diffraction pattern of a fiber of DNA, comprehensive work was carried out in the second half of the 20th century to relate DNA’s structure to the solution properties observed using hydrodynamic and scattering techniques. In addition to the iconic image of the double-helical geometry, the following geometrical parameters were derived: a helical pitch of 34 Å, with planar base pairs (bp) stacked with a separation of 3.4 Å, so that each turn contains 10 bp, and a diameter of about 20 Å. An important question about the structural determinations obtained using X-ray diffraction crystallography has always been whether the results obtained from a biomacromolecule contained in such a crystal or paracrystalline fiber under X-ray irradiation—particularly the values of the geometric parameters—correspond well to the structure in a physiological, aqueous solution. Furthermore, as DNA is a long, high-molecular-weight macromolecule, its biological function must depend on how much the properties and behavior of DNA in solution are determined by the overall conformation and dynamics of such a long polynucleotide chain. Thus, experimental determination and structural modeling of DNA was a central topic in biophysics during the second half of the 20th century and beyond, as described in well-known monographs [78,79] and textbooks [80,81].

Establishing relationships between solution properties and structural properties requires a model whose properties can be obtained using theoretical and computational calculations. The theory for increasingly complex and detailed models is obviously increasingly difficult, and the cost of computation increases with the number of model elements. The need for more or less coarse-grained models is then evident. The double helix of double-stranded DNA, dsDNA, demonstrates a slight bending flexibility. Small oligonucleotides—say, up to 20 bp—are too short to display bending curvature, and present a rigid, straight, rod-like morphology. Additionally, longer fragments look like a bending rod or a curved filament; an illustrative example is shown below. For sufficiently long DNA—say, over 300 bp—dsDNA will present a typical flexible random-coil conformation. Earlier investigations on structure–property relationships in dsDNA focused on medium-to-high-molecular-weight dsDNA, such as those from monodisperse fragments, calf tymus, lambda bacteriophage, etc. Some of the approaches have been flexible-chain, bead-and-spring Rouse–Zimm models [26,27] (as described above) modified to account for chain stiffness [82,83].

Bead-and-spring models have some limitations when representing dsDNA, including the inability to represent its transversal structure and thickness. This is relatively unimportant for native DNA with a molecular weight usually over $10^{6}$ Da, but gains importance for shorter fragments. In much shorter DNA, although still flexible, their conformational statistics depart from the fully random coil represented by the Rouse–Zimm chains. Instead, the DNA model widely adopted from the 1970s was based on the Kratky–Porod [84] worm-like chain model [78,79]. This model regards a polymer as a continuous semiflexible filament with a uniform, circular cross-section of constant thickness/diameter and variable curvature, which is gauged using a parameter, the persistence length, *P*, that indicates the degree of local flexibility and depends, therefore, on the nature of the material being represented, with larger values for stiffer polymers. The conformation of the chain is determined using the ratio of the contour length *L* to *P*; for a small L/P, the chain looks like a weakly bending rod. A straight rod corresponds to the limit L/P→0, while for a large L/P value, the conformation is a random coil. With this model, even the shortest DNA oligonucleotides can be represented by a rod with length *L* and diameter *d*.

Still, the uniform, cylindrical cross-section of the worm-like model still neglects the double-helical geometry of dsDNA. In a more detailed model, the elements would represent small molecular units arranged in a double helix. Such a model is described in the next section, and the following section will consider the worm-like model.

### 5.1. A Double-Helical Model for dsDNA

**Figure 3:**
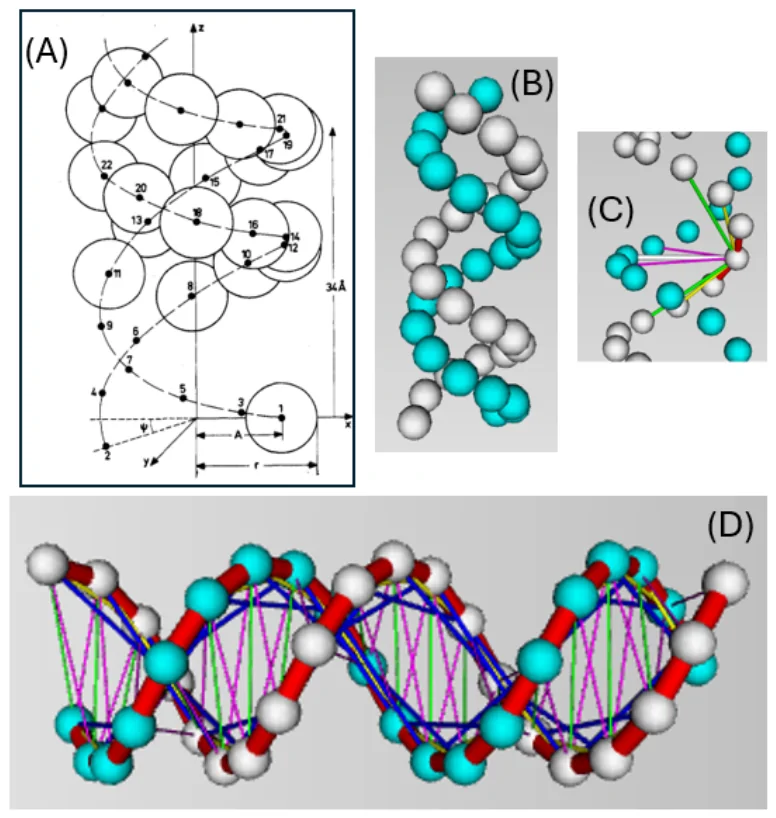
(**A**) Construction of a rigid touching-beads model of the double helix used to calculate the overall properties of dsDNA [32,86]. (Reprinted with permission from García de la Torre and Horta [32] ©1976 American Chemical Society.) (**B**) View of the resulting straight double-helical model for a nucleotide with $N_{bp}=20$ bp. (**C**) Connectivity in the flexible model. Every bead, *i*, is joined to (a) its two first neighbors i±1 (red), two second neighbors i±2 (yellow), and two third neighbors i±3 (green), in its strands; (b) its pair in the opposite strand, $j=i+N_{bp}$ or $j=i-N_{bp}$ (white), and the two first neighbors of the strand, j±1 (pink). (**D**) View of the 20 bp elastic network in the flexible model in a straight conformation. According to the helix geometry in (A), the radius of the tangent beads that represent the nucleotide bases is $\sigma =\left(1/2\right)\sqrt{0.516A^{2}+p^{2}}$, where *A* is the radius of the helical backbone in Å, and p=3.4 Å is the helical pitch. The connectors are stiff Fraenkel springs, as in Equation (7), with equilibrium length $l_{e}=2\sigma$ and a large force constant $H=200k_{B}T/l_{e}^{2}$. This value ensures that the relative rms fluctuation, from Equation (8), is $\delta_{l}^{*}=0.015$, so that $\delta_{l}$ is only 1.5% of $l_{e}$.

The possibility of predicting the solution properties of DNA using a relatively realistic model, representing the double-helical bead model with just one element per nucleotide—what is, at the present time, named a “coarse-grained model”—was put forward in a work co-authored by one of us 50 years ago. In that work, a double helix with 10 beads per turn in each strand and a pitch of 3.4 Å per base pair was modeled. Beads were tangential to their two neighbors (Figure 1 in the paper by García de la Torre and Horta [32]; Figure 3A below) and the diameter of the double helix was considered an adjustable parameter.

In those early years, experimental data on short oligonucleotides were available only for polydisperse samples—fragments produced by DNA ultrasonic or shear degradation—and the hydrodynamic treatment of rigid bead models was based on the primitive Kirkwood equation [85]. In the 1980–90s, the accurate calculation of translational and rotational diffusion coefficients of short oligonucleotides with 8 to 20 base pairs [86] was facilitated by the availability of experimental data for monodisperse samples of oligonucleotides prepared with restriction enzymes [87] and the advanced hydrodynamic theory and computational models implemented in our HYDRO program [11] for rigid structures. The results for translational and rotational diffusion coefficients were in good agreement with experimental data [87].

The very slight local flexibility of the double helix is not sufficient for representation in the overall diffusion coefficients of such small oligomers, which behave as rigid particles, but their internal dynamics are indeed detected using torsional twisting flexibility [87] and overstretching under a tensional force [88].

These phenomena were successfully replicated using Brownian dynamics simulations with a primitive version of SIMUFLEX [58,89]. The coarse-grained bead-per-residue model was based on the rigid, straight one in which a net of relatively stiff springs was introduced, so that the network is locally quasi-rigid, as it should be for short oligonucleotides, but still slightly flexible so that long, native DNAs behave as less or more flexible filaments. The double-helical hydrodynamic model for dsDNA is illustrated in Figure 3. From the trajectories obtained via Brownian dynamics simulations (see Video S1), the translational and rotational diffusion coefficients of oligonucleotides up to 20 bp were predicted and found to be very close, as expected, to those obtained previously for the fully rigid model [86]. Furthermore, the dynamics simulation allowed us to analyze the internal dynamics manifested in torsion [90,91], stretching [88], etc.

**Figure 4:**
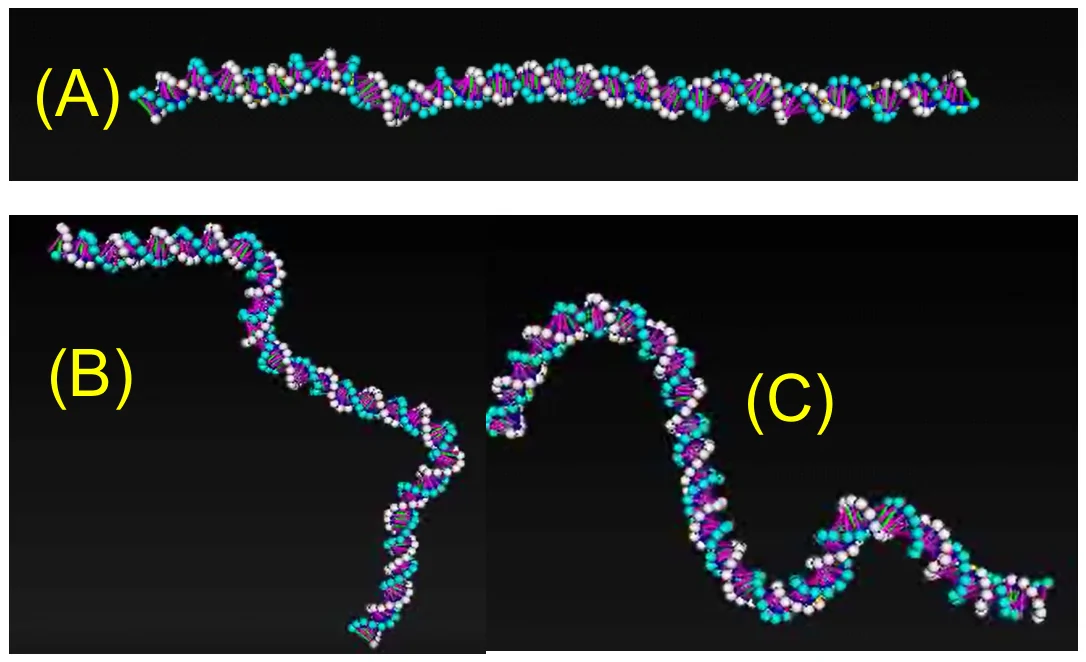
Conformation snapshots of a dsDNA fragment with 148 base pairs along a Brownian dynamics simulated trajectory. (**A**) The simulation started from a straight conformation of the double helix. (**B,C**) After a sufficient number of steps, the memory of the initial state is lost and, throughout the rest of the simulation, the conformation of the dsDNA shows the typical worm-like aspect.

Thus, in our coarse-grained bead-per-residue model, the network of springs is locally quasi-rigid, as it should be for short oligonucleotides, but still slightly flexible so that longer DNAs behave as less or more flexible filaments. For over 100 bp, the simulation shows the onset of flexibility, manifested by the fluctuating, curved conformation. From the simulated trajectories, using our ancillary program VisualBeads, it was possible to record movies showing the Brownian internal motion of moderately large DNA fragments, such as the video included in the Supplementary Information for a 148 bp long dsDNA (see Video S2). This length is sufficient for the molecule to adopt a worm-like aspect as illustrated by the movie. In Figure 4, we present two snapshots of instantaneous conformations of that fragment.

### 5.2. Worm-like DNA: From 8 de 200,000 Base Pairs

Calculations based on a double-helical, bead-per-nucleotide model require a computing time that notably increases with the size of the DNA segment. The number of elements in the model—two per base pair—is proportional to the molecular weight, *M*, as $N=2M/M_{bp}$, where $M_{bp}\approx 600–660$ Da. For instance, for calf thymus DNA with M≈12 million Dalton ($1.2\times 10^{7}$ Da), the double-helical model would contain $N\approx 4\times 10^{4}$ elements, and the simulation with ordinary resources is unfeasible.

As indicated above, the well-known model used to calculate solution properties of DNA is the worm-like chain (WC). The flexibility parameter is the persistence length, *P*, and the conformation of a chain of contour length *L* is determined by the ratio L/P. The contour length is simply related to the molecular weight by the mass-per-unit length, $M_{L}=M/L$, which is determined by the local chemical structure of the macromolecule. For L/P<<1, the model is a rigid straight rod of length *L* and diameter *d*, while towards the other limit, when L>>P, the conformation is a random coil. In this direction, it was common in early papers to express the degree of flexibility by the Kuhn statistical length, $L_{K}$ [92,93], which can be regarded as the contour length of a polymer chain beyond which it behaves as a fully flexible coil and is simply twice the persistence length, $L_{K}=2P$ [94,95]. The hydrodynamic properties depend on an additional parameter, the hydrodynamic diameter *d*, which is the effective diameter of the uniform, circular cross-section of the worm-like filament, which is to be interpreted as a measure of the thickness of dsDNA, including a hydration layer of water molecules and some counterions.

**Figure 5:**
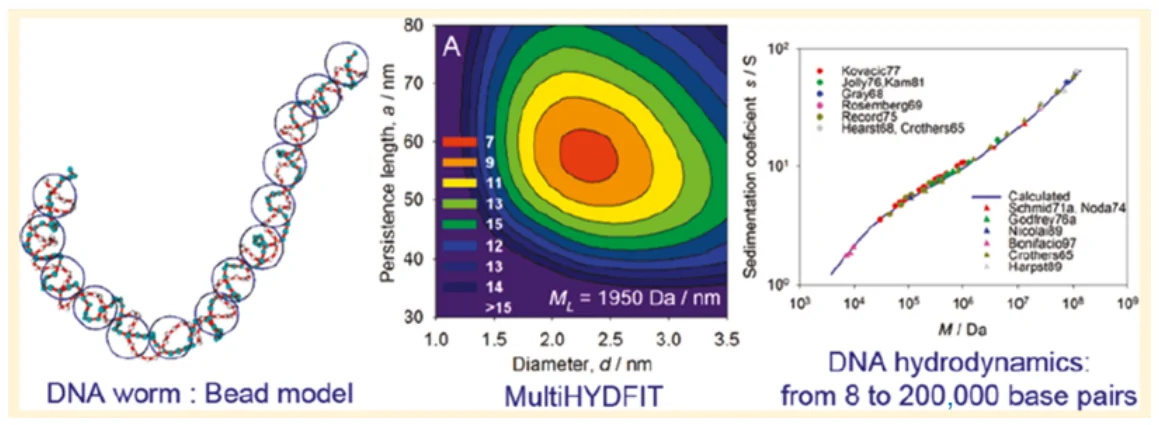
(**Left**) Worm-like chain of touching beads, superimposed to the worm-like double-helical model that is replaced. (**Center**) Searching for the best fitting WC parameters: plot of the typical percent deviation in equivalent radii, 100Δ, as a function of *P* and *d*. (**Right**) Determining the goodness of the fit: based on experimental and calculated values of the sedimentation coefficient along a range of five decades in molecular weight. Similar plots of *D*, [η], and $R_{g}$, highlighting the high agreement with our results, can be found in ref. [101]. (Reprinted with permission from Amorós et al. [101] ©2011 American Chemical Society.)

Yamakawa and Fujii [96,97] were the first to attempt to provide a theoretical description of the hydrodynamic properties of dsDNA in a wide range of sizes and determine the values of the WC model parameters. Some restrictions and approximations in hydrodynamic theory affected the numerical results for the fitted parameters or the range of treatment validity. Later on, Hagerman and Zimm [98] proposed to adopt a discrete version of the worm-like model proposed by Schellman [99,100], which was a partially flexible worm-like string of beads instead of a worm-like cylinder, and they computed rotational diffusion coefficients via rigid-body Monte Carlo simulation. More recently, we improved on this approach by employing advanced bead-modeling hydrodynamics [73] and Monte Carlo algorithms, in a comprehensive simulation study on the worm-like model [101]. The model is a chain of *N* touching beads, as shown in Figure 5, the diameter of which is equal to the distance between centers *b*. Instead of the worm-like line, the skeleton is a polygonal of *N* straight segments of length *b*, so that the contour length is L=Nb.

In our mechanical model (a simple version based on the Allison–McCammon [102,103] model), the segments are treated as stiff Fraenkel springs (Equation (7)) with equilibrium length $l_{e}\equiv b$ and a large spring constant, $H=100k_{B}T/l_{e}^{2}$, so that the fluctuation in *ℓ* around $l_{e}$ is very small. Then, like in the Schellman chain, the bending flexibility is described by a quadratic angular potential (Equation (11)), with an equilibrium angle corresponding to a straight, unbent conformation, $\alpha_{e}=\pi$, and an angular spring constant related to the persistence length, $K_{\alpha }=k_{B}Tb/P$. Furthermore, the bead size corresponds to the diameter of the worm-like cylinder, equal to the hydrodynamic diameter, *d*, such that the volume of the worm is the same as that of the string of beads [98]; this criterion entails that $b={\left(3/2\right)}^{1/2}d$.

Thus, we obtained computational results for the translational coefficients (diffusion *D* and sedimentation *s*), intrinsic viscosity [η], and radius of gyration $R_{g}$ of chains across the whole range of parameters for the WC model [101]. This set of numerical values is a data base for the evaluation—via interpolation/extrapolation—of the properties as a function of *P*, *d*, and *L* or *M* and $M_{L}$ with our new programs WORM1 and WORM2 (in the EllipCylinWORM package) [9]. Then, we compiled a large set of values for the dsDNA properties, covering an extensive size range $M=5\times 10^{3}$–$1.2\times 10^{8}$ Da, i.e., from 8 to 200,000 base pairs (from an oligomer with only 8 bp to T2 phage DNA). The whole set of equivalent radii, including hydrodynamic radii ($R_{H,f}\equiv a_{T}$ from *s* or *D*, $R_{H,\eta }\equiv a_{\eta }$ from [η]) and the radius of gyration ($a_{G}\equiv =\sqrt{5/3}R_{g}$), was analyzed via global least-squares fitting of 147 data points $\left(a_{X},M\right)$, with *X* = *T*, *I*, and *G*. This fit consists of minimizing the function

(14) $$100\Delta =100{\left[\sum_{X=T,G,I}{\left(\frac{a_{X}\left(calc\right)-a_{X}\left(exp\right)}{a_{X}\left(exp\right)}\right)}^{2}\right]}^{1/2}$$

which represents a typical percent deviation from experimental values, where the sum over X=T,G,I is extended to all available values of $a_{T}$, $a_{I}$, and $a_{G}$. This procedure is implemented in our program MultiHYDFIT [68,104,105], which can perform a global analysis of numerous data of multiple properties in a given model (WC in this case). The results for the WC model of dsDNA were the following: a=560±20 Å, d=23±1 Å and $M_{L}=195\pm 4$ Da/Å, with a typical percent deviation of 100Δ=6.6%. Our result for *P* is in the range observed in previous studies with a much more reduced data set, and our results for *d* and $M_{L}$ are in extraordinarily good agreement with the composition and geometric values corresponding to the Watson–Crick double helix of the B-form of dsDNA, which is shown in Figure 5. Taking 3.4 Å as the rise per base pair (ratio $h=L/N_{pb}$) and $M_{bp}=660$ Da/Å as the average base-pair molecular weight, the mass-per-unit length should be $M_{L}=M/L=M_{bp}/h=660/3.4=194$ Da/Å. Furthermore, as the phosphorus atoms are placed at a distance of about 10 Å from the central helical axis, the diameter of the bare DNA double helix would be about 20 Å. With an additional hydration layer of water molecules and sodium counterions, one would expect a hydrodynamic diameter *d* of 22–24 Å.

Our ability to obtain these parameters, in such high agreement with the geometry of the Watson–Crick double helix, using only readily accessible experimental measurements of properties like *D*, [η], $R_{g}$, and *s* is proof of an important concept: Hydrodynamic and other dilute-solution properties are valuable sources of information regarding macromolecular structure, with regard to not only overall conformation but also local chemical structure.

### 5.3. Anomalous Sedimentation of Very Long DNAs

About 100 years after the invention of analytical ultracentrifugation (AUC), it currently remains as possibly the most comprehensive technique for characterizing macromolecular structures, as well as dynamics and interactions in solution [106,107], and it has played a central role in biophysical studies on DNA [78,79]. Sedimentation velocity experiments have revealed a very important hydrodynamic property: the sedimentation coefficient, *s* [80,81]. The dependence of *s* on the molecular weight *M* and the conformation of the macromolecular solute are important sources of structural information.

In the 1970s, it became apparent that the sedimentation coefficient of very long double stranded dsDNAs—with *M* beyond $10^{8}$ Da—deviated remarkably from the trend of the *s*-*M* dependence, with values that decreased with increasing rotor speed, ω [108] (for conventional macromolecules, *s* does not depend on ω). Zimm and others presented a theory for the rotor-speed dependence of the observed *s*, based on the approximate hydrodynamic theories available at the time [109,110,111,112].

**Figure 6:**
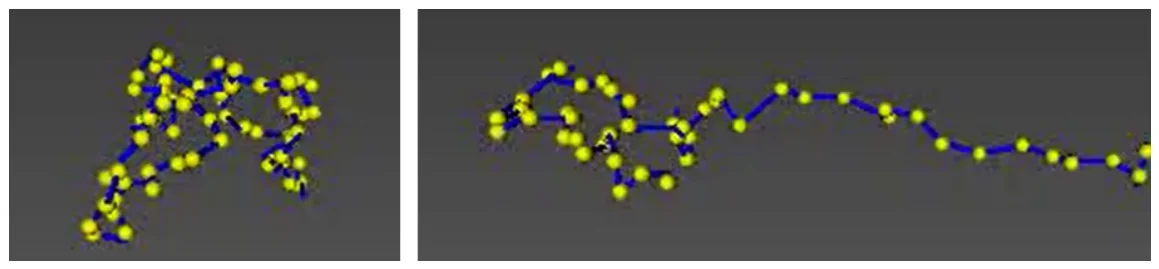
Snaptshots generated by our program VisualBeads from the trajectory of a Brownian dynamics simulation of anomalous sedimentation by García de la Torre et al. [114]. (**Left**) Initial, random-coil conformation of a self-avoiding bead-and-spring model with N=60 beads, modeling a molecule of the DNA of T2 bacteriofage with $M=1.2\times 10^{8}$ Da. (**Right**) A “tadpole” conformation after 20 s under centrifugation with a rotor speed ω=45,000 rpm at a radial distance to the rotor r≈6.5 cm. The centrifugal field goes from left to right.

An important contribution toward solving this problem was made more recently by Schlagberger and Netz [113], who carried out a Brownian dynamics simulation of a model chain under the action of a centrifugal force. The simulation clearly showed the origin of the rotor-speed effect on long, flexible, random-coil chains. We have reproduced their findings using our SIMUFLEX programs, which incorporate the possibility of external forces. In a suitable bead-and-spring model chain with self-avoiding excluded-volume internal interaction, the beads, with molecular weight $M_{1}=M/N$ and mass $M=M_{1}/N_{A}$ ($N_{A}$ is the Avogadro number) experience forces $F=\alpha_{b}m$, where $\alpha_{b}=\omega^{2}r\left(1-\bar{v}\rho \right)$ is the centrifugal acceleration, *r* is the radial position of the bead, $1-\bar{v}\rho$ is the buoyancy factor, and then $\bar{v}$ and ρ are the specific volume of the macromolecular solute and the solvent density, respectively. Using BROWFLEX, we simulated the trajectories of such model (details given in our previous publication [114]). Then, ANAFLEX, first of all, provided visualizations of the chain evolution under the centrifugal field (see Figure 6), which confirmed the striking fact found by the aforementioned authors: the coil suffers an elongation and adopts an extended conformation that resembles a tadpole, with a coiled head and a tail attached along the radial direction, towards the bottom of the centrifuge cell.

**Figure 7:**
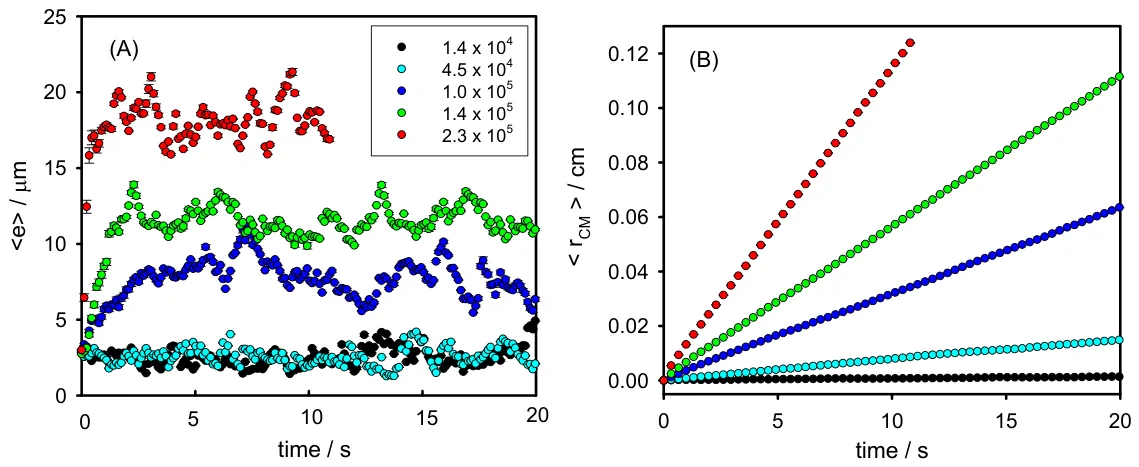
(**A**) Time evolution of the chain extension from the initial value for the model of T2 DNA, under the indicated values of the rotor speed, ω, in rpm. (**B**) Time evolution of the radial position of the center of mass for the same ω (same legend as (**A**)). The plotted values are the sample average for a large number of individual molecules $r_{CM}$. (Reprinted with permission from García de la Torre et al. [114] ©2010 John Wiley and Sons.)

ANAFLEX also provides valuable information about the time evolution of several data points for each of the many molecules in the simulation sample. One data point of interest for characterizing the stretching caused by external flows or fields is the extension, *e*, defined as the longest intramolecular (bead-to-bead) distance in an instantaneous conformation. The time course of the sample average of this quantity during the first 20 s in the centrifugal field is shown in Figure 7. While at a low rotor speed, ω, the extension remains nearly unchanged; at higher speeds ω, the chain suffers a sudden extension at the beginning of centrifugation, immediately reaching a stationary, slightly fluctuating value. A video file created with our ancillary program VisualBeads (see Video S3) displays the evolution of the model chain immediately after the start centrifugation, from its initial random coil to the stretched, tadpole-shaped conformation.

**Figure 8:**
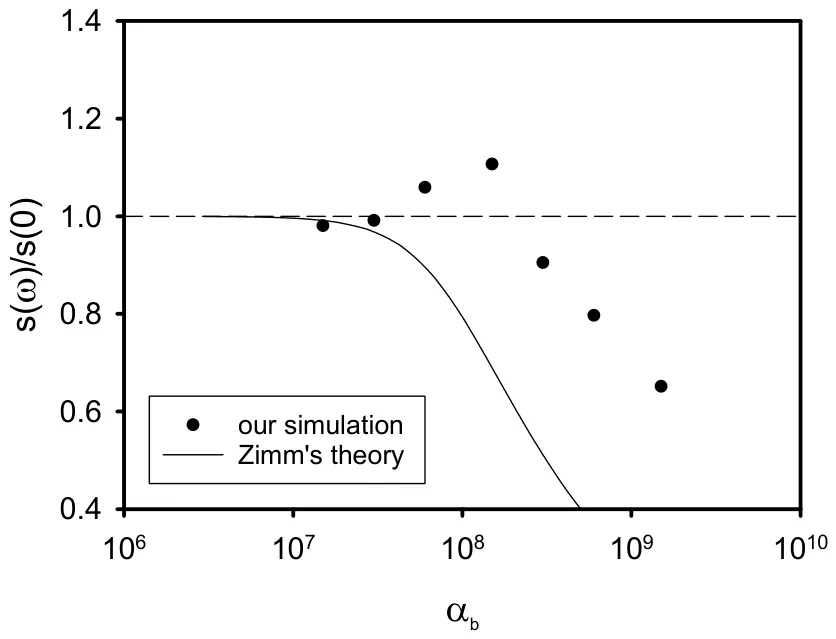
Dependence of the sedimentation coefficient of T2 DNA, normalized to the value of the initial random-coil conformation, on the rotor speed. The dashed line indicates the value for the random-coil conformation. Numerical values obtained from our BD simulation and the plot of the corrected Zimm theory [109,112]. (Reprinted with permission from García de la Torre et al. [114] ©2010 John Wiley and Sons.)

Another of the many functions provided by ANAFLEX is the extraction of a file with the position coordinates of the center of mass, $r_{CM}$, of the simulated particle at a series of selected time points along the Brownian trajectory. Figure 7 displays the radial position of the center of mass, $r_{CM}$, during the first 20 s. The sedimentation velocity of the molecule is $v=dr_{CM}/dt$, i.e., the slope of the *v* vs. *t* plots. The sedimentation coefficient is defined as the ratio of the sedimentation velocity to the centrifugal acceleration, $s=v/\alpha_{b}$. As shown in Figure 8, the simulation results show the decrease with rotor speed, but the numerical values depart from Zimm theory. It is well known that the departure is caused by the various approximations in his formalism. Thus, the BD methodology, and tools like SIMUFLEX, shows its adequacy for describing complex aspects of intramolecular dynamics.

## 6. Intrinsically Disordered Proteins

Over the past two decades, research on proteins that lack a fixed, well-defined structure and organization in their natural state, exhibiting a degree of disorder, has become increasingly important in biomedical sciences, and particularly in biophysics. Understanding their role, for example, in neurodegenerative diseases requires thorough knowledge of their structure and dynamics, and in this regard, the study of their properties and behavior in solution is clearly relevant.

Some intrinsically disordered (ID) proteins have polypeptide chains with a completely disordered conformation in their native state; although they exhibit notable internal structural peculiarities, proteins with such chains present an overall conformation and behavior typical of a flexible chain or random coil and are considered one class of ID proteins.

**Figure 9:**
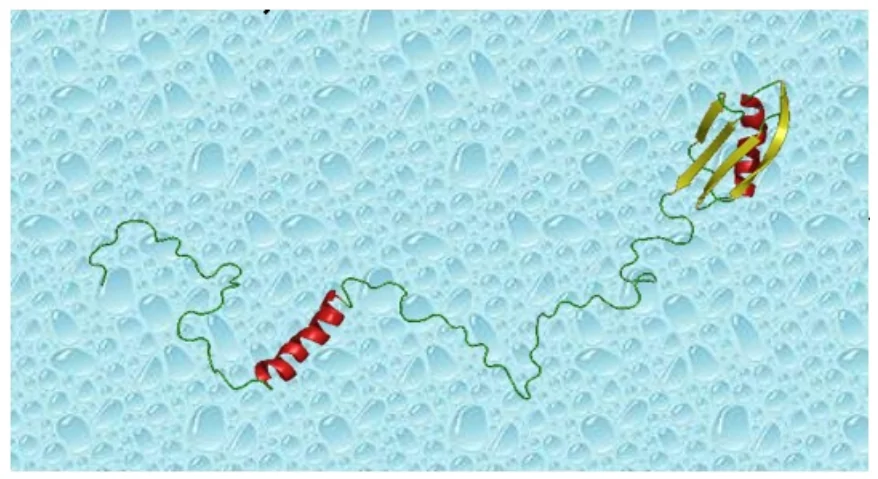
Typical aspect of a partially disordered protein: the chimeric construct is indicated in the text.

There is another class of partially disordered proteins, in which parts of the chain are ordered and compact, like globular proteins (these are intrinsically disordered regions, IDR). In other regions, however, the chain is unfolded, forming linkers between the globular domains or tails that hang from them. An illustrative example of this situation is shown in Figure 9. The heterogeneous conformations of proteins with IDRs is certainly a primary reason for their peculiar functional role [115], which is apparently related to their dynamics in solution [116]. Thus, knowledge of their hydrodynamic properties and dynamics is of evident importance, due to not only their dynamics but also their characterization. Furthermore, these aspects constitute an interesting challenge in macromolecular hydrodynamics modeling because they have the extraordinary feature of containing both rigid and flexible domains.

Thus, ID proteins with IDRs constitute a challenging application of Monte Carlo and Brownian dynamics tools. In our work on this topic [59,117], we wanted to design a minimalistic, highly simple coarse-grained model with the minimum requirements to achieve adequate predictions of the properties in solution and the dynamic behavior of these proteins using our tools for flexible macromolecules: MONTEHYDRO and SIMUFLEX. Indeed, such simplicity would also result in significant computational savings in the simulations.

**Figure 10:**
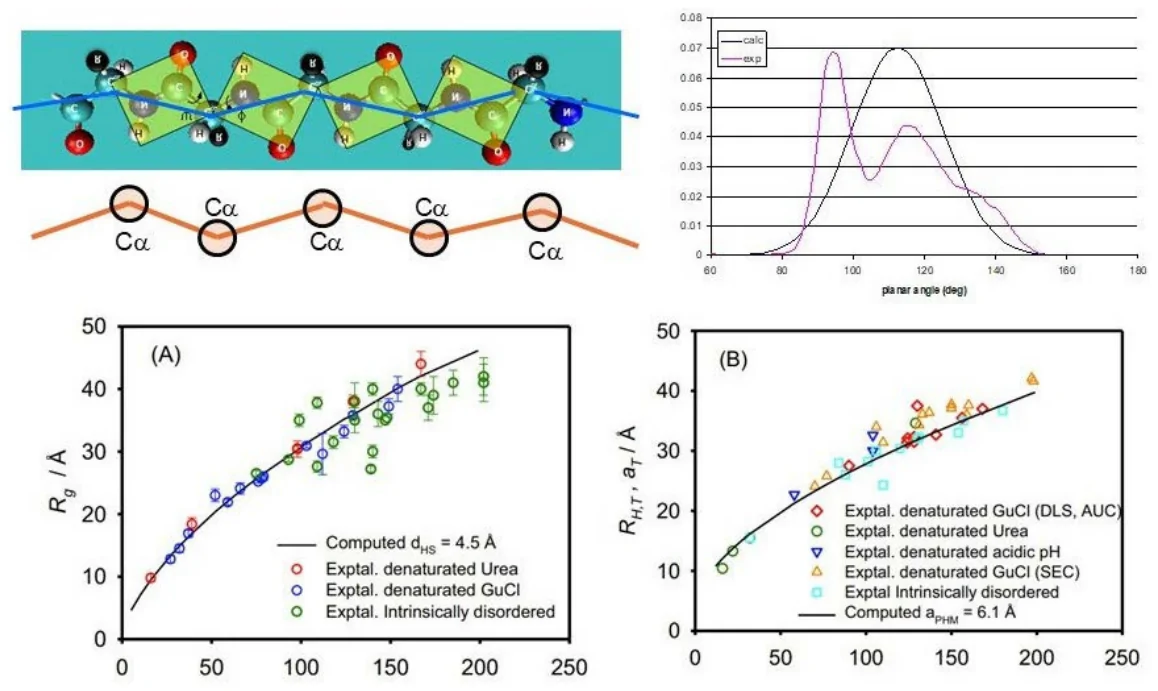
**Top**: Scheme presenting the polypeptide chain as a chain of virtual $C_{\alpha }$–$C_{\alpha }$ bonds of length 3.8 Å. Statistics of the $C_{\alpha }$–$C_{\alpha }$–$C_{\alpha }$ angles for the determination of parameters in the quadratic angular potential [117]. **Bottom**: Experimental and calculated values of the (**A**) radius of gyration and (**B**) hydrodynamic radius of unfolded—denatured and intrinsically disordered—proteins [8].

Our minimalistic coarse-grained residue-level model was built considering the following criteria:

1. The model has only one element (bead) per aminoacid, which are all the same size, with a hydrodynamic bead radius of 6.1 Å. This value was chosen to be the same as the bead radius in our residue-level hydrodynamic rigid-bead model employed for proteins in HYDROPRO version 2011 [12].

2. Given that the peptide group is nearly planar and rigid, the polypeptide chain backbone in the disordered domains—i.e., linkers and tails—is represented by a chain of $C_{\alpha }$–$C_{\alpha }$ virtual bonds, which in our mechanical model are stiff Fraenkel springs (Equation (7)) with an equilibrium length as long as the virtual bond, $l_{e}=$ 3.8 Å [118]. As the bond must be quasi-rigid, in the mechanical model, a quite small fluctuation is required. In accepting a ±5 % fluctuation, i.e., $\mathrm{relative}\;\mathrm{rms}\equiv \delta_{l}^{*}=0.05$, and then using Equation (8), the value of the Hooke constant should be $H=k_{B}T/\left[l_{e}{\left(\delta_{l}^{*}\right)}^{2}\right]=4.3\times 10^{-4}$ dyn/cm.

3. The other short-range interaction in the flexible polypeptide chain is that associated with bending the angle between neighbor bonds, α. A statistical study by Kleywegt [119] on the bending angle in random-coil regions in ordered proteins resulted in the distribution shown in Figure 10. In order to employ the simple quadratic potential, Equation (11), in the simulations, the experimental distribution was replaced with a best-fitting Gaussian distribution (as it should be for the Boltzmann exponential associated with Equation (11)). The mean value was found to be $\alpha_{e}=112^{\circ }$, and the force constant was $K_{\alpha }=2.4\times 10^{-12}$ erg/${\mathrm{rad}}^{2}$.

4. Torsional interactions, involving four consecutive virtual bonds, were also considered by Kleywegt. Although certainly necessary for other purposes, we assumed that they would not be essential for the statistics of the virtual-bond chains.

5. Long-ranged pair-wise interactions are absolutely indispensable, especially those corresponding to steric, attractive/repulsive excluded-volume interactions between beads not involved in previous short-ranged interactions (bond and bond–angle interactions). As indicated above, an efficient choice is the hard-sphere potential, which limits interbead distances to $r_{ij}<d_{HS}$ (Equation (13)). For the parameterization of this kind of interaction, for both disordered and ordered regions, we adopted the value in the classical Gō model [120,121] (*vide infra*): $d_{HS}$ = 4.5 Å. This potential is fully operational in MC simulation, but in BD, which manages expressions for forces, discontinuous potentials are not feasible. Thus, as proposed in the Gō model, we employed a continuous repulsive potential, as frequently used in Gō-like models:

(15) $$V_{ij}=\epsilon_{2}{\left(\sigma_{2}/r_{ij}\right)}^{12}$$

Using a set of experimental data for the $R_{g}$ of urea-denatured proteins [122], it was found via MC simulation that the hard-sphere potential reproduced them very well [117]. Also, BD simulations with the repulsive exponential, Equation (15), with the Gō model parameter $\sigma_{2}=4$ Å and $\epsilon_{2}$ in the range 1–4 Kcal/mol yielded results very close to those obtained via MC simulation, finding the best fit for $\epsilon_{2}\approx$ 2 Kcal/mol.

As anticipated by Tozzini [123], we confirm that the minimalistic model for the unfolded polypeptide chain, constructed with the five conditions described in the previous paragraphs, can be used to predict, with sufficient satisfaction, a conformational and hydrodynamic property, the radius of gyration $R_{g}$ and the hydrodynamic (Stokes) radius $R_{h}$ respectively, of chains of up to 200 residues, as shown in Figure 10. The plots for both properties vs. chain length show the agreement between a compilation of experimental data [124,125,126,127] and simulation results. Certainly, this analysis could be improved through the use of more data, longer proteins, etc., but at this point, it offers complete confidence in the use of this modeling scheme for the flexible linkers and tails of partially disordered proteins.

Then, for the globular, quasi-rigid domains, our model incorporated aspects from the widely appreciated Gō model [120,121], which was initially devised for simulating protein folding and has since been extended to a variety of (hydro)dynamic topics [128,129]. The folded polypeptide backbone is represented as a chain of identical beads joined by virtual bonds as mentioned above in paragraphs 1 and 2, with the following additional features:

6. Like in the case of flexible domains, a potential is assigned to the bond angles (paragraph 3) even though it is now stronger: the equilibrium value in Equation (11) is determined using the PDB coordinates, and a larger potential constant $K_{\alpha }$ = 40 Kcal/mol restricts the variability of the bond angles much more. In addition, a torsional potential is now include in the model (for more details, see ref. [59]).

7. The main concept underlying this Gō-like model is that of *native contacts*. Some pairs of $C_{\alpha }$ elements, although appreciably separated along the sequence, are considered to be native in the sense that they dictate the folding of the chain and are responsible for the quasi-rigid 3D structure of the ordered domain. Among the various algorithms for determining the native contact pairs [130], we employed the simple and widely used CSU (Contacts of Structural Units) program of Sobolev et al. [131]. For the elements in a contact pair, a special pair-wise distance potential dependent was employed:

(16) $$V\left(r_{ij}\right)=\epsilon_{1}\left[5{\left(\frac{\sigma_{1}}{r_{ij}}\right)}^{12}-6{\left(\frac{\sigma_{1}}{r_{ij}}\right)}^{10}\right]$$

Meanwhile, for all of the other non-native pairs, the same potential and parameters as those employed for the disordered, flexible domains were employed (paragraph 5). Our implementation of the Gō-like model follows essentially that of other simulation studies on protein folding [128,129]. Parameter values and more details can be found in our previous work [59]. All of the force fields and potentials are program modules in MONTEHYDRO and BROWFLEX.

**Figure 11:**
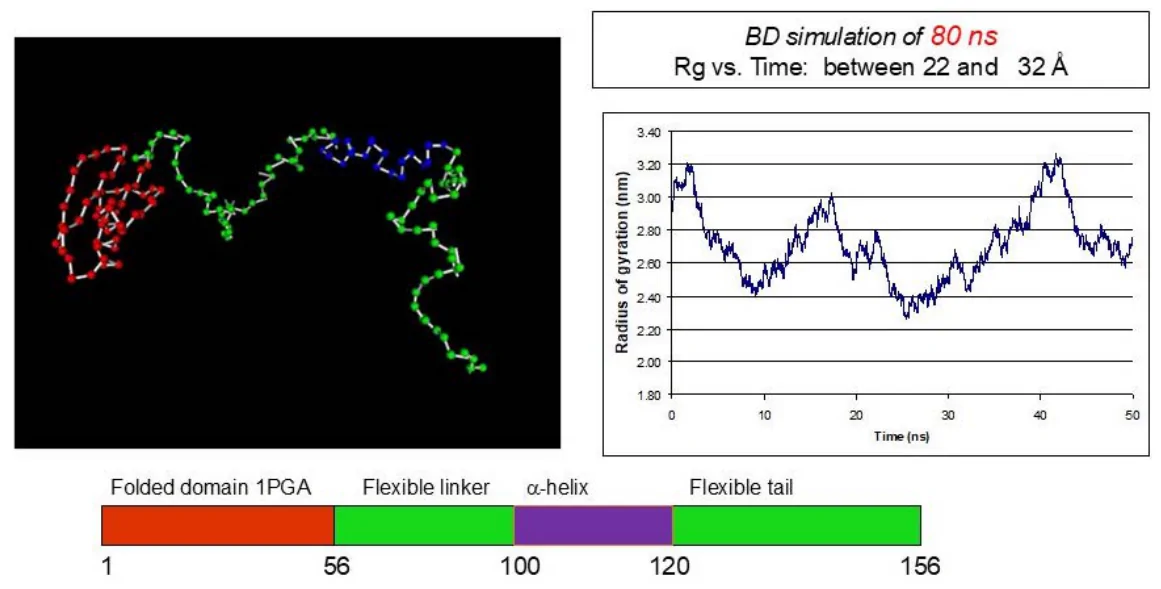
Structure of a partially disordered protein employed for testing and benchmarking purposes (previously displayed in Figure 9). Domain I is an ordered, C-terminal globular domain, the structure of which was taken from the immunoglobulin-binding domain of protein G (1PGA.pdb) with 56aa [137]. Domain II is a disordered, flexible linker with 44 glycine residues. Domain III is another ordered, α-helical domain with 25 amino acids with the same structure as that of the peptide pleurocidin (1Z64.pdb27) [138]. Domain IV is a C-terminal flexible tail with 31 glycine residues. The evolution of the instantaneous radius of gyration along 80 ns shows that the protein domains remain stable, as shown in the snapshot of an instantaneous conformation. A movie of the whole trajectory is available in the Supplementary Information (see Video S4).

The simulation and analysis protocols were tested with a chimeric protein constructed ad hoc, with two ordered domains—one globule and one helix—and two unfolded flexible-chain domains—a linker and a hanging tail—as shown in Figure 11. A Brownian dynamics simulation was carried out using BROWFLEX, and the trajectory was processed with ANAFLEX. A trace of the protein radius of gyration, $R_{g}$, fluctuates between the expected limits. A snapshot of an instantaneous conformation is shown. Furthermore, the whole trajectory was visualized as a video generated with our program MovieBeads, which shows the real-time internal Brownian motion of the protein, with the ordered domains keeping their size and shape stable along the whole trajectory.

As mentioned already, the same mechanical model (i.e., same topology and force field) is common for both MONTEHYDRO and SIMUFLEX. MONTEHYDRO provides rigorous results for conformational properties, like $R_{g}$, and scattering-related quantities, like intramolecular distances, the scattering form factor, etc., and provides well-approximated values for single-valued hydrodynamic coefficients, which have been found to be in close agreement with the rigorous hydrodynamic results from SIMUFLEX. Beyond the macromolecular characterization based on single-valued properties, $R_{g}$, *D* and *s*, [η], etc., [125,127,132,133,134], SIMUFLEX also allows the study of more complex experimental properties that reflect the internal structure and dynamics of flexible macromolecules determined using techniques like static and dynamic light, X-ray, and neutron scattering [125,127,133,134] as well as nuclear magnetic resonance (NMR) [135,136]. Currently, these techniques are essential for the study of intrinsically disordered proteins, and they complement coarse-grained modeling and simulation methods well [132].

In our previous studies [8,59], we applied this computational scheme to a variety of partially disordered proteins to predict solution properties and compare the results with experimental data. The results for single-valued properties, particularly the radius of gyration and the sedimentation coefficient listed in Table 1, were very satisfactory. Considering the structural complexity of these macromolecules, and the complexity involved in their modeling and simulation procedures, the quality of the agreement is rewarding. In ref. [59], the reader may find equally satisfactory results for even more complex properties, like the angular dependence of small-angle X-ray scattering and the sequence dependence of the T1/T2 ratio of NMR relaxation times.

## 7. “Almost Natural” Synthetic Polymers: Dendrimers

Following the presentation of the simulation programs and their applications to two macromolecular systems of great biological relevance, double-stranded DNAs, and intrinsically disordered proteins, we chose dendrimers as an example from the field of synthetic polymers. As detailed below, these polymers have many characteristics in common with globular proteins such as an almost spherical globular shape, high internal density, and activity localized mainly on its surface, and they are also of great importance in biosciences, as drug delivery agents.

Dendritic polymers (dendrimers) are hyperbranched molecules with a completely regular branching structure. Starting from an internal core, there is a certain number of branches (their functionality, *F*); at the terminal end of each branch, there is a node from which two branches, identical to the previous ones, emerge as a new generation, and so on. Each branch acts as a monomeric unit of the polymer; for a dendrimer of *G* generations, the number of branches is $F(2^{G}-1)$ (with G=0 being the core, and G=1 the generation emerging from the core) so that their molecular weight increases exponentially with *G*. Since their synthesis was described by Tomalia and coworkers [139], dendrimers have been the subject of intensive experimental, theoretical, and computational research. Even from the point of view of basic polymer physics, they present a very peculiar feature: beyond some number of generations, their intrinsic viscosity decreases with increasing molecular weight [140]. This is just one of the many aspect that has been explained via simulation [141,142]. In our group, we developed a scheme based on the concept of a mechanical model simulated by either Monte Carlo or Brownian dynamics, with the tools described in this review.

**Figure 12:**
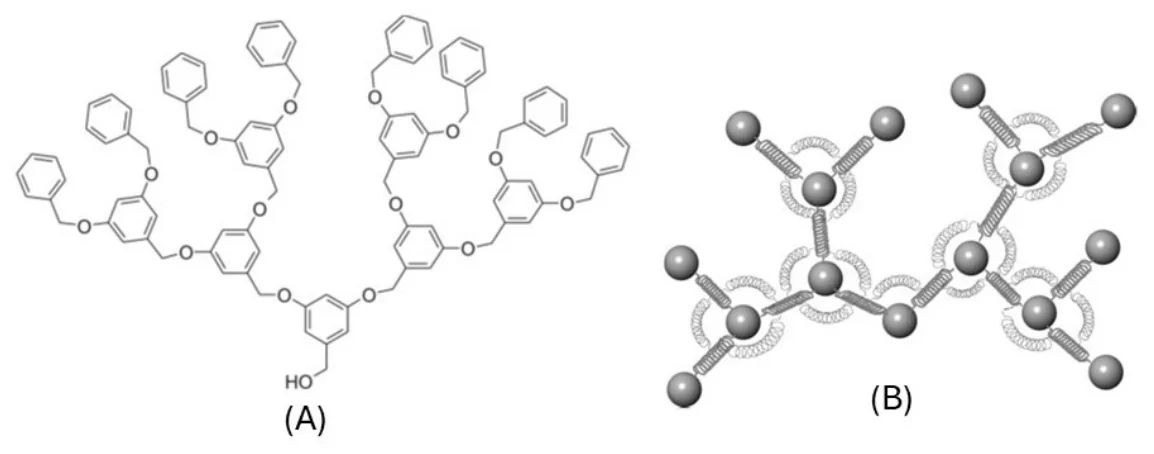
(**A**) Chemical structure of a mono-PBzE dendrimer with G=3, and (**B**) its corresponding coarse-grained, mechanical model. (Reprinted with permission from Del Río et al. [52] ©2009 American Chemical Society.)

Let us consider a simple dendrimer, mono-polybenzyl ether (mono-PBzE), which has a bifunctional core (F=2), as shown in Figure 12. The bead-and-spring model consists of beads centered at the nodes and connecting springs between them. Each branch is a monomeric unit represented by a spring and its terminal bead. For the spring length, we employed a variant of the general FENE-Fraenkel spring with three parameters: force constant $H_{HF}$, equilibrium length, $l_{e}$, and maximum length, $l_{max}$. The potential energy of the spring is given by

(17) $$V_{conn}\left(l\right)=-\frac{1}{2}H_{HF}l_{max}^{2}\ln\left(1-\frac{l^{2}}{l_{max}^{2}}\right)-\frac{1}{2}H_{HF}l_{max}l_{e}\ln\left(\frac{l_{e}+l}{l_{e}-l}\right)$$

and for the bending angle between any pair of adjacent springs, the simple angular potential, Equation (11), with parameters $K_{\alpha }$ and $\alpha_{e}$ is adequate.

**Figure 13:**
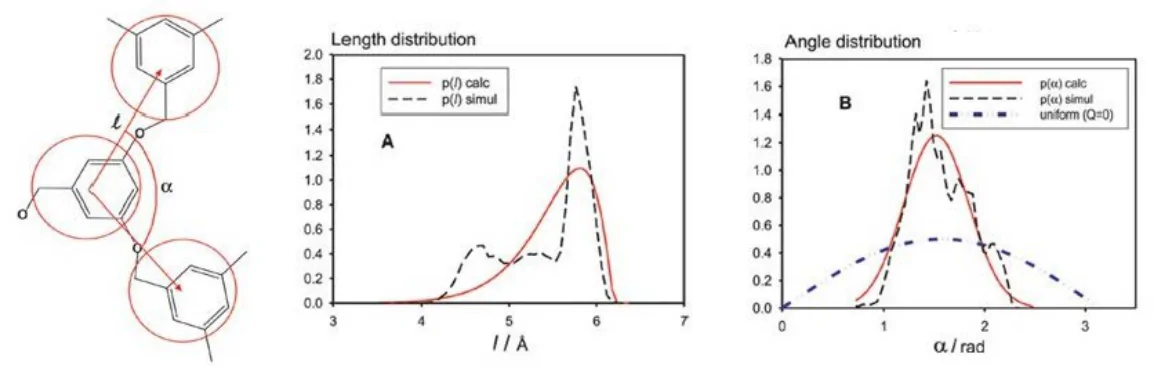
Chemical structure of a trimer of mono-PBzE used to fit spring and angular parameters. An atomistic MD simulation was carried out with commercial software HYPERCHEM using the AMBER force field. From instantaneous values of *ℓ* and α extracted from the simulated trajectories, the value of $l_{max}$ and the distributions p(l) and p(α) were determined. These distributions where regarded as the Boltzmann exponentials, $p\propto \exp(-V/k_{B}T)$, of the potentials V(l), V(α) in Equations (17) and (11), respectively. By fitting the simulated distribution to the Boltzmann statistics, the mentioned model parameters were obtained (plots (**A**,**B**)). (Reprinted with permission from Del Río et al. [52] ©2009 American Chemical Society.)

Parameterization is performed using a typical multiscale approach. The bead size is determined from the atomic structure of the monomer by generating conformations through atomistic molecular dynamics (MD) simulation and calculating the hydrodynamic radius using the HYDROPRO program [12]. MD simulations of a trimer (Figure 13) are also carried out to determine the parameters for the distribution of the spring connectors (i.e., that of the monomer’s end-to-end length), obtaining its mean and maximum values, which are used to parameterize the corresponding spring (see Figure 13). These simulations also yield the distribution of angles between adjacent branches, from which the parameters of the angular springs are determined. Note that this is a properly multiscale scheme, as there are no adjustable parameters in the mechanical model; all parameters for MONTEHYDRO and SIMULFLEX are obtained through simulations at a smaller scale of the elements that comprise it, using prior knowledge.

Table 2 compares the results calculated for mono-PBzE dendrimers through the MONTEHYDRO simulation of the mechanical model with the parameters resulting from the multiscale scheme, with experimental data. The typical (rms) deviation from experimental data is just a few percent; in absolute values, it is 0.7 Å for $R_{g}$—roughly the size of a hydrogen atom—and 0.2 ${\mathrm{cm}}^{3}$/g for [η], which is less than typical experimental error. The numerical agreement is very satisfactory, but one should note the importance of the number of diverse aspects involved in the multiscale simulation.

**Figure 14:**
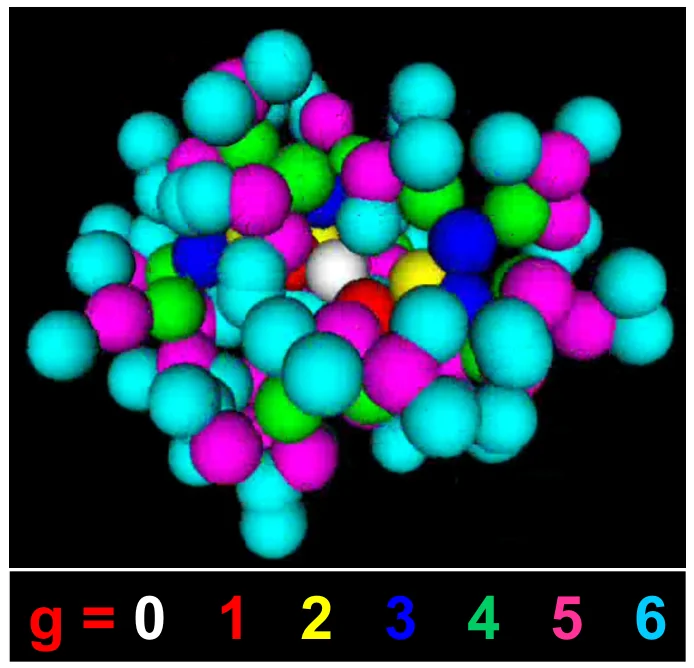
An instantaneous conformation of the dendrimer mono-PBzE of six generations, where each generation, g, is represented with a different color. A snapshot of the BROWFLEX-simulated BD trajectory presented in Video S5 is shown, where the size of the beads is real.

Among the many relevant results of recent studies on dendrimers, one indicates that their interior is quite dense (close to that of globular proteins), as observed experimentally using SAXS [143,144,145] and verified via simulation [51,52,146], although they have spaces in which foreign molecules can be accommodated. This is why they are considered excellent vectors for drug delivery [147,148]. This porous internal structure is not static. In our previous studies, BD simulation with SIMUFLEX has shown remarkable internal mobility of the branches, as seen in the visualization generated using VisualBeads (see Video S5). Figure 14 shows an instantaneous conformation obtained from the trajectory recorded in file dendrimero1X.wmv (Video S5). This mobility is important for the encapsulation of a drug molecule, as it allows for the spontaneous release of the molecule. SIMUFLEX enables the simulation and visualization of how a molecule initially located inside the dendrimer (very close to the core) can reach the outside (see Video S6). As has been recently recognized, computational modeling is a very valuable tool in the design of drug delivery systems based on nanoparticles [149,150].

## 8. Concluding Remarks

Dilute-solution properties are of utmost importance in the study of macromolecules, and they serve as a fundamental basis for their characterization in practice. For example, techniques based on solution (intrinsic, Newtonian) viscosity—which were crucial to the pioneering, classic studies that established the macromolecular concept [6,7]—are widely used today to characterize the size and conformation of large synthetic and biological molecules, employing modern methodologies such as microfluidics [151].

In this article, we first briefly present the theoretical background underlying the Monte Carlo (MC) and Brownian dynamics (BD) simulation methods implemented in our programs. It is worth noting that the treatment of the fundamental hydrodynamic interaction effect lies at the core of SIMUFLEX and MONTEHYDRO, mirroring the approach used in the HYDRO suite programs. Indeed, they all share the same structural design and are similar in their handling of the 3N×3N diffusion tensor, D (Equations (1)–(3); Section 2.1). In fact, as an alternative to this BD methodology, based on the Ermak–McCammon algorithm [13] and its subsequent modifications [17,18,19,21], other different methodologies have been developed, including Langevin Dynamics (LD) simulation [152,153], the Lattice Boltzmann method (LB) [154,155], Dissipative Particle Dynamics (DPD) [156,157], and Nonequilibrium Molecular Dynamics (NEMD) [158,159].

Therefore, there are, admittedly, other theoretical schemes and stochastic simulation software packages that cover the dynamics of systems not covered by our methods, such as biomolecular association, diffusion in concentrated solution or crowded environments, cellular transport, and so on. Even some general-purpose, multi-method programs capable of describing some aspects of the behavior of biomacromolecules in solution (e.g., CafeMol [160] or LAMMPS [161]) may not be fully optimized for this specific purpose.

A detailed comparison of our programs with others is beyond the scope of this article. As mentioned in the Introduction, since our work focuses on the properties, structure, and dynamic behavior of macromolecules in dilute solution, our programs—particularly BROWFLEX—are dedicated strictly to this scenario. Consequently, they rigorously internally implement the effects of intramolecular interactions—such as hydrodynamic interaction and electrostatic forces, and model proper features of biophysical systems like unequal or/and overlapping beads. Also, as previously noted, MONTEHYDRO and SIMUFLEX have been developed with an emphasis on simplicity, aiming to make them user-friendly and accessible to users with minimal computer skills. The design—based on text files for input and output, precompiled executables, and clear, instructional user manuals—has been retained here, following the successful approach used in other programs from our group (like HYDRO++ [73], HYDROPRO [12,69], HYDRONMR [162], etc.). In our experience, they have proven valuable even for teaching purposes, serving as tools for computational exercises, student projects, and the like.

As examples of the use of our theoretical and computational applications in which MONTEHYDRO and SIMUFLEX have been employed, we present practical examples of their use and results. The main body of the applications has focused on what could be considered the quintessence of biomacromolecules: double-stranded DNA, dsDNA. During the 1960s and early 1970s, theoretical advances in the physical chemistry of macromolecules focused, in addition to synthetic polymers, on elucidating the structure and dynamics of dsDNA observed experimentally through hydrodynamic and scattering properties in solution. The state of the art at that time is reflected in the monograph by Bloomfield, Crothers, and Tinoco [78], and the theoretical aspects are covered in the work of Yamakawa [15,96,97,163,164].

With the advent of computational resources in the late 1970s and throughout the 1980s, a plethora of simulation studies emerged using conventional MC and BD simulation methods, with the latter based on the Ermak–McCammon algorithm and its modifications. Our procedures, applied to the simulation of dsDNA in solution, have continued along these lines, incrementally incorporating the theoretical and computational advances already mentioned. Thus, other applications could be implemented such as the simulation of the behavior under intense elongational flows, including the so-called molecular individualism [74,165,166,167] and double-strand fracture [168,169,170].

It was not until well into the 1980s that the aforementioned alternative schemes emerged and were also applied to dsDNA. A recent advancement in the field is the work of de Pablo and coworkers, who implemented an advanced coarse-grained model in LAMMPS. This is the 3SPN.2 (3-Site-Per-Nucleotide) model [171,172], in which each nucleotide is modeled by three beads representing the phosphate group, the sugar group, and the base group, enabling the study of large-scale biophysical processes in double- and single-stranded DNA (under this nomenclature, our coarse-grained double-helical model proposed in 1976 [32] and subsequent work for dsDNA [58,86] and ds-motifs in RNA [173] would be 1SPN, i.e., one site/bead per nucleotide).

Thus, the double-helix structure of dsDNA has been extensively studied using Monte Carlo and dynamic methods. Within the field of DNA biophysics, a recent challenge has emerged concerning how such studies can be carried out for DNA-based macromolecular complexes, with chromatin as the paradigmatic case. Formalisms from polymer science [174,175] are currently available for designing coarse-grained models on which to perform dynamic simulation using Brownian dynamics [176,177] or other methods [178]. Having these techniques is crucial for modeling normal physiological functions as well as altered states, as it has been very recently demonstrated [179]. The tools that we have developed support coarse-grained models of any type; the force field of the mechanical model includes many diverse modules that enable the simulation of macromolecular complexes as complicated as chromatin.

## 9. Computer Program Repository

All of the computer programs featured in this review are freely available, including detailed user guides and several examples of input and output files, from our web site https://leonardo.inf.um.es/macromol (accessed on 17 August 2026).

## Figures and Tables

**Table 1:** Results for some partially disordered proteins. ^*a*^ Total number of aminoacid residues, the sequence of domains (from N-terminus to C-terminus), indicating the number of residues and globular (G) type, or flexible linker or tail (L). ^*b*^ Properties: sedimentation coefficient in S units, radius of gyration in Å. From ref. [8] and references therein.

Protein	Topology ^*a*^	Properties ^*b*^
pX, Sendai virus	95 = 42(L) + 53(G)	$R_{g}$ = 29.7 or 25 (exp.), 26.7 (calc.)
ZipA	306 = 163(L) + 143(G)	s = 2.0 (exp.), 2.1 (calc.)
S4	336 = 193(g) + 50(L) + 193(G)	$R_{g}$ = 39.8 (exp.), 38.3 (calc.)
MMP-12	365 = 155(G) + 30(L) + 180(G)	$R_{g}$ = 25 (exp.), 30.3 (calc.)
BTK	659 = 169(G) + 51(L) + 55(G)	$R_{g}$ = 50 (exp.), 49 (calc.)
	+ 8(L) + 96(G) + 22(L)	s = 3.3 (exp.), 3.6 (calc.)

**Table 2:** The radius of gyration and intrinsic viscosity of mono-PBzE dendrimers of *G* generations are listed. *N* is the number of monomeric branch units, as well as the number of elements in the model. The experimental values and results were obtained from the MONTEHYDRO simulation performed by del Río et al. [52].

			*Experimental*		*Calculated*	
G	M, g/mol	N	$R_{g}$, Å	[η], ${\mathrm{cm}}^{3}$/g	**$R_{g}$, Å**	**[η], ${\mathrm{cm}}^{3}$/g**
2	745	7		3.7	6.8	3.5
3	1595	15	9.9	4.5	9.2	4.3
4	3292	31	11.6	5.2	11.8	4.9
5	6687	63	13.5	5.3	14.7	5.2
6	13,479	127		5.0	18.2	5.3
*rms percent difference*					6.5%	4.9%
*rms difference*					0.7	0.2

## Data Availability

No new data were created or analyzed in this study. Data sharing is not applicable to this article.

## Supplementary Materials

The following supporting information can be downloaded at: https://www.mdpi.com/article/10.3390/ijms27177791/s1.
