# Supplementary material for: Simulating Dilute-Solution Properties and Behavior of Flexible Macromolecules: A Review of Brownian Dynamics, Monte Carlo Methods, and Computational Tools (SIMUFLEX and MONTEHYDRO) with Applications to Biomacromolecules and Selected Synthetic Polymers
Source: Int J Mol Sci. 2026 Aug 31;27(17):7791. doi: 10.3390/ijms27177791 (PMC13565817; doi:10.3390/ijms27177791)
Supplement: Supplementary file 1 [file ijms-27-07791-s001.zip › Supplementary Information.pdf]

Supplementary Information

We provide movies created for the analysis of Brownian dynamics simulations (simulated via BROWFLEX, analyzed via ANAFLEX), which produced a graphic file processed with VisualBeads, from which a video file was generated in .wmv (Windows Media Video) format.

- Video S1: File DNA20bp.wmv shows the trajectory of a double-helical model of a 20-base-pair dsDNA oligonucleotide (from Huertas et al. [59]).
- Video S2: File DNA148bp.wmv shows the trajectory of a double-helical model of a 148-base-pair fragment of dsDNA (from Huertas et al. [59]).
- Video S3: File AnomSed.wmv shows the behavior of a long dsDNA molecule during sedimentation under a strong centrifugal field.
- Video S4: File quimera.wmv shows the trajectory of our model for the chimeric protein employed in a test case in our study (from Amoros et al. [60]).
- Video S5: File dendrimero1X.wmv shows the trajectory of a model for a 6th generation dendrimer (from Del Rio et al. [53]). The snapshots in Figure 14 were taken from this simulation.
- Video S6: File VideoDendriEncaps.wmv displays the time evolution of a model for a 5th generation dendrimer (from Del Rio et al. [53]) into which a foreign, guest molecule (red particle) was initially encapsulated. This represents a proof-of-concept tool for drug delivery research.
